# Correlates of Perceived Stress Among Community-Dwelling Older African Americans Without Dementia

**DOI:** 10.1093/geroni/igab046.1001

**Published:** 2021-12-17

**Authors:** Crystal Glover, Ana Capuano, Robert Wilson, David Bennett, Lisa Barnes

**Affiliations:** 1 Rush University Medical Center, Chicago , Illinois, United States; 2 Rush, Chicago, Illinois, United States; 3 Rush University, Chicago, Illinois, United States; 4 Rush University Medical Center, Chicago, Illinois, United States

## Abstract

The purpose of this study was to identify correlates of perceived stress among older African Americans without dementia. We grouped correlates into four levels – environmental, sociocultural, behavioral, and biological – guided by the National Institute on Aging’s (NIA) Health Disparities Research Framework. We performed a cross-sectional data analysis with the Minority Aging Research Study using ordinal logistic regression analyses. Participants were 722 African Americans without dementia [mean age = 73.61 years (SD=6.33)]. Correlates from environmental (e.g., larger life space), sociocultural (e.g., larger social network size), behavioral (e.g., more purpose in life), and biological (e.g., higher global cognition) levels were associated with lower odds of having greater levels of perceived stress. Perceived stress was associated with correlates from every level. Future research is needed to examine how changes in these correlates may be related to perceived stress in older African Americans.

